# Metformin sensitizes anticancer effect of dasatinib in head and neck squamous cell carcinoma cells through AMPK-dependent ER stress

**DOI:** 10.18632/oncotarget.1628

**Published:** 2014-01-07

**Authors:** Yu-Chin Lin, Meng-Hsuan Wu, Tzu-Tang Wei, Yun-Chieh Lin, Wen-Chih Huang, Liang-Yu Huang, Yi-Ting Lin, Ching-Chow Chen

**Affiliations:** ^1^ Graduate Institute of Pharmacology, National Taiwan University College of Medicine;; ^2^ Department of Oncology, National Taiwan University Hospital;; ^3^ Department of Internal Medicine and; ^4^ Department of Pathology, Far-Eastern Memorial Hospital;

**Keywords:** AMPK, ER stress, EGFR, dasatinib, PDK4

## Abstract

Head and neck squamous cell carcinoma (HNSCC) is an important endemic disease in Taiwan with aggressive course and dismal outcome. Dasatinib is a Bcr-bl and Src kinase inhibitor that has potential against HNSCC. We recently disclosed that EGFR degradation is critical for dasatinib-induced apoptosis. Here, we further demonstrate that AMPK-dependent ER stress is responsible for this event. Dasatinib induced ER stress which mediated EGFR degradation in a c-cbl-dependent manner. AMPK activation induced by dasatinib might be due to ATP decrease through the up-regulation of pyruvate dehydrogenase kinase 4 (PDK4). Furthermore, activation of AMPK by metformin sensitized dasatinib-induced in vitro and in vivo anti-cancer effect. The correlation of AMPK activation and EGFR expression was seen in HNSCC cells and human tumor specimens. Our results disclose that AMPK-dependent ER stress plays a crucial role in the anti-cancer effect of dasatinib in HNSCC and further activation of AMPK by metformin might enhance dasatinib efficacy.

## INTRODUCTION

Endoplasmic reticulum (ER) is the primary site for protein synthesis, folding, and trafficking. Metabolic stress impairs protein processing capacity to accumulate nascent proteins leading to ER stress [1]. Unfolded protein response (UPR) can restore homeostasis by halting protein translation, enhancing protein degradation, and increasing molecular chaperon production [2]. If ER stress is persistent and unresolved, UPR will switch pro-survival signaling to proapoptotic to remove damaged cells [2]. The UPR is regulated by three ER intermembrane transducers: PKR-like ER kinase (PERK), inositol requiring 1 (IRE1), and activating transcription factor 6 (ATF6) [1]. PERK phosphorylates initiation factor 2α (eIF2α) to attenuate protein synthesis, and eIF2α can also regulate CHOP expression to mediate apoptosis [3]. ER stress resulted from rapid proliferation-induced nutrient deficiency and hypoxia in cancer cells is compensated by pro-survival UPR [4]. Thus, ER stress might be a target for cancer treatment.

AMP-activated protein kinase (AMPK) is a serine/ threonine protein kinase activated by energy deficiency to promote cellular catabolism of nutrients to gain energy [5]. Epidemiological studies show that patients with metabolic syndrome have decreased AMPK activity and increased cancer mortality [6-7]. Metformin, an AMPK activator, has been reported to reduce the incidence of cancer in type 2 diabetes patients [8-9]. These indicate that AMPK activation might have an anti-cancer effect. Furthermore, AMPK activators are reported to exert anti-cancer effect through ER stress induction [10-11], suggesting the association between AMPK activation and ER stress.

Head and neck squamous cell carcinoma (HNSCC) is a worldwide disease composed of oral, pharyngeal, and laryngeal squamous cell carcinoma closely associated with alcohol, betel nut, cigarette, and human papilloma virus (HPV) infection [12]. In Taiwan, the incidence of oral cancer is higher than that of Western countries because of betel nut chewing. Epidermal growth factor receptor (EGFR) is a well-known oncoprotein belonging to receptor tyrosine kinase family which transduces signalings through Ras-MAPK and PI3K-Akt pathways to promote cellular growth, proliferation, and survival [13]. EGFR is highly expressed in HNSCC and its expression is correlated with advanced stage of disease and poor prognosis [14]. Cetuximab is an anti-EGFR monoclonal antibody approved as the first molecular-targeted therapy for HNSCC [15]. Despite the incorporation of cetuximab, the prognosis of advanced HNSCC remains poor [16]. Thus, developing new agents are urgent.

Dasatinib is a kinase inhibitor of Bcr-abl and Src approved to treat chronic myeloid leukemia (CML) [17]. Since Src is an oncoprotein closely associated with solid tumor proliferation and invasion [18], dasatinib is expected to exert activity against HNSCC. However, few HNSCC patients benefit from dasatinib in clinical trials despite consistent Src inhibition [19], indicating that mechanism beyond Src inhibition might be responsible for dasatinib efficacy. Identification of the molecular mechanism might further improve dasatinib activity. Our recent work demonstrates that EGFR degradation is critical for dasatinib-induced apoptosis [20]. In the present study, we further showed that this effect is mediated by AMPK-dependent ER stress. Up-regulation of pyruvate dehydrogenase kinase 4 (PDK4) might be responsible for dasatinib-induced ATP decrease and AMPK activation. The association of AMPK activation and EGFR expression was seen in HNSCC cells and human specimens. Furthermore, activation of AMPK by metformin sensitized dasatinib-induced anti-cancer effect in vitro and in vivo. Our results disclose that AMPK-dependent ER stress plays a crucial role in the anti-cancer effect of dasatinib in HNSCC and further activation of AMPK by metformin might enhance dasatinib efficacy.

## RESULTS

### ER stress mediated dasatinib-induced EGFR degradation and apoptosis

**Figure 1:**
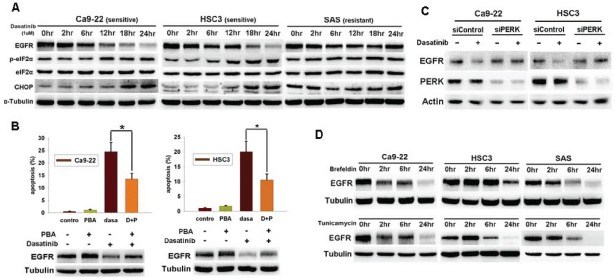
Dasatinib induced ER stress and EGFR degradation in HNSCC cells (A) The effect of dasatinib on ER stress and EGFR expression. The expression of EGFR, p-eIF2α, eIF2α, and CHOP was evaluated in HNSCC cells treated with dasatinib (1uM) for indicated time. (B) The effect of 4-phenyl butyric acid (PBA) on dasatinib-induced EGFR degradation and apoptosis in Ca9-22 (left) or HSC3 (right) cells. *Upper*, SubG1 analysis of HNSCC cells treated with indicated drugs for 48hrs. Column, mean (n=3); bar, standard deviation; *, p<0.05 by paired Student's t-test. *Lower*, the expression of EGFR after treated with indicated drugs for 24hrs. (C) The effect of PERK knockdown on dasatinib-induced EGFR degradation. Cells were transfected with control or EGFR siRNA for 72 hours and then treated with dasatinib (1uM) for 24 hours. (D) The effect of ER stress on EGFR expression. Cells were treated with brefeldin-A (5ug/ ml) or tunicamycin (2ug/ml) for indicated time. The expression of proteins was evaluated by Western blotting. Representative of three independent experiments was shown.

We recently find that dasatinib-induced EGFR degradation occurs in sensitive but not resistant cells, and c-cbl-dependent lysosome pathway is responsible for this effect [20]. Because c-cbl was activated by Src to facilitate EGFR endocytosis and degradation [18], dasatinib-induced Src inhibition might inhibit c-cbl to impair EGFR trafficking and degradation. Therefore, mechanism other than Src inhibition might be responsible for dasatinib-induced c-cbl activation and EGFR degradation. We found that dasatinib induced eIF2α phosphorylation and CHOP expression in sensitive Ca9-22 and HSC3 cells (IC_50_ 0.45uM and 0.78uM, respectively) but not resistant SAS cells (IC_50_ > 10uM) (figure 1A), indicating that dasatinib inducted ER stress in sensitive cells. 4-phenyl butyric acid (PBA), an ER stress inhibitor, attenuated dasatinib-induced EGFR degradation and apoptosis (figure 1B). Knockdown of PERK by RNA interference also reversed dasatinib-induced EGFR degradation (figure 1C). These results indicated the involvement of ER stress in dasatinib-induced EGFR degradation and apoptosis. To further investigate the effect of ER stress on EGFR degradation, ER stress activators, brefeldin-A or tunicamycin were used. They both decreased EGFR expression (figure 1D).

**Figure 2:**
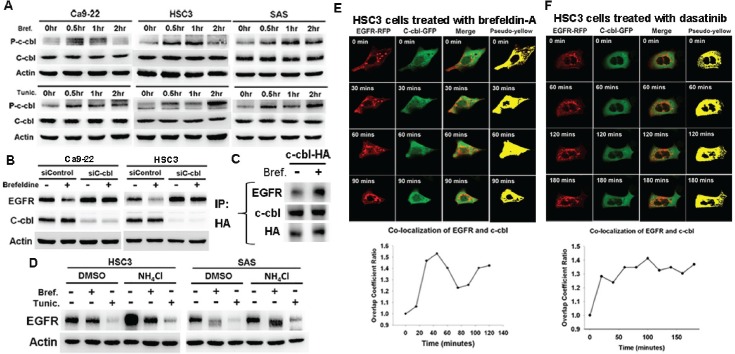
C-cbl-lysosome pathway mediated ER stress-induced EGFR degeradation (A) The effect of ER stress on c-cbl activation. Cells were treated with brefeldin-A (5ug/ml) or tunicamycin (2ug/ml) for indicated time. The expression of p-c-cbl, and c-cbl was evaluated. (B) The effect of c-cbl knockdown on brefeldin-A-induced EGFR down-regulation. Cells were treated with control or c-cbl siRNA and then treated with brefeldin-A for 24 hours. The expression of EGFR and c-cbl was evaluated. (C) The effect of ER stress on the association of c-cbl and EGFR. Cells were transfected with c-cbl-HA plasmid and treated with brefeldin-A for 2 hours. Cell lysates were immunoprecipitated with anti-HA antibodies. The immunoprecipitates were blotted with EGFR and c-cbl. (D) The effect of lysosome inhibitor NH_4_Cl on brefeldin-A or tunicamycin-induced EGFR down-regulation. Cells were treated with indicated drugs for 24 hours, and the expression of EGFR was evaluated. (E,F) The localization of EGFR and c-cbl under ER stress. HSC3 cells were co-transfected with EGFR-RFP and c-cbl-GFP. The localization of EGFR (red) and c-cbl (green) of live cells treated with brefeldin-A (E) or dasatinib (F) was recorded by time-lapse confocal microscopy (*upper panel*), and the co-localized signal was pseudo-colored in yellow (lane 4). The ratio of co-localization was calculated by Zen software (Carl Zeiss) (*lower panel*)

C-cbl-lysosome pathway is well-known for EGFR degradation [18, 21]. C-cbl phosphorylation (p-c-cbl, Y774) was increased by brefeldin-A or tunicamycin (figure 2A), and knockdown of c-cbl reversed brefeldin-A or tunicamycin-induced EGFR down-regulation (figure 2B). To further investigate the association between EGFR and c-cbl, co-immunoprecipitation analysis showed the increased association of c-cbl and EGFR by brefeldin-A (figure 2C). On the other hand, lysosome inhibitor, NH_4_Cl, attenuated brefeldin-A or tunicamycin-induced EGFR down-regulation (figure 2D). These results indicated that c-cbl-dependent lysosome pathway is associated with ER stress to induce EGFR degradation.

To further evaluate the association of EGFR and c-cbl under ER stress, time-lapse confocal microscopy was performed to record the localization of EGFR and c-cbl. The co-localized EGFR-RFP and c-cbl-GFP is pseudo-colored in yellow and the ratio is calculated. The co-localization between EGFR (red) and c-cbl (green) was increased by brefeldin-A (figure 2E and video file 1) or dasatinib (figure 2F and video file 2), which also implies the involvement of c-cbl in ER stress-induced EGFR degradation.

### The role of AMPK in dasatinib-induced ER stress and EGFR degradation

**Figure 3:**
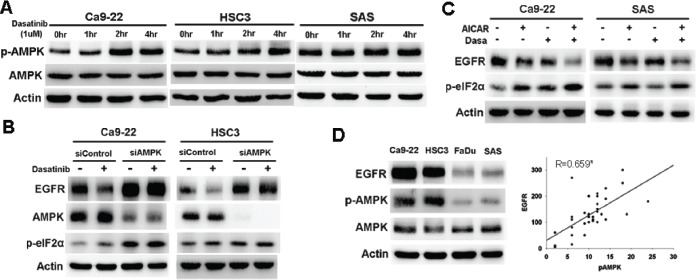
AMPK activation mediated dasatinib--induced ER stress and EGFR degradation (A) The effect of dasatinib on AMPK activation. Cells were treated with dasatinib (1uM) for indicated intervals. The expression of p-AMPK and AMPK was evaluated. (B) The effect of AMPK knockdown on dasatinib-induced EGFR degradation and ER stress. Cells were treated with control or AMPK siRNA and then with dasatinib for 24 hours. (C) The effect of AMPK activation on dasatinib-induced EGFR degradation. Cells were treated with dasatinib with or without AICAR (10uM) for 24 hours. The expression of EGFR p-eIF2α, and AMPK was evaluated. (D) The correlation between p-AMPK and EGFR expression. *Left*, the expression of EGFR, p-AMPK, and AMPK in HNSCC cells. *Right*, the correlation of p-AMPK and EGFR expression in resected human specimens. Pearson's correlation coeffcient=0.659; *, p<0.01

Activation of AMPK has been reported to induce ER stress [10-11]. Our previous work also revealed that statin induced ER stress through AMPK activation [22]. AMPK activation (p-AMPK, T172) was induced by dasatinib in sensitive Ca9-22 and HSC3 but not resistant SAS cells (figure 3A). Knockdown of AMPK reversed dasatinib-induced EGFR degradation and eIF2α phosphorylation (figure 3B). Addition of Compound C, an AMPK inhibitor, also attenuated dasatinib-induced EGFR degradation (figure S1A). These indicated that AMPK played a role in dasatinib-induced EGFR degradation.

To further evaluate the effect of AMPK activation on ER stress and EGFR degradation, AMPK activators, AICAR or 2-DG was used. Both of them induced phosphorylation of c-cbl and eIF2α and down-regulation of EGFR (figure S1B). Furthermore, AICAR enhanced dasatinib-induced EGFR degradation and eIF2α phosphorylation in sensitive Ca9-22 cells (figure 3C, *left*). In resistant SAS cells, EGFR and p-eIF2α were not affected by dasatinib. However, AICAR in combination with dasatinib induced EGFR degradation and eIF2α phosphorylation in resistant SAS cells (figure 3C, *right*). These results suggest that AMPK activation is crucial for dasatinib-induced ER stress and EGFR degradation.

### Correlation between phosphor-AMPK and EGFR in HNSCC cells and patients

Since AMPK knockdown induced EGFR up-regulation (figure 3B, compare lane 1 and 3), the relationship between AMPK and EGFR expression in HNSCC cells and human specimens was evaluated. Phosphor-AMPK but not AMPK was correlated with EGFR expression in HNSCC cells (figure 3D, *left*). Immunohistochemical (IHC) staining of 38 tumor specimens from HNSCC patients showed that the expression of p-AMPK or EGFR was heterogeneous (figure S1C and S1D). The IHC scoring of p-AMPK was positively correlated with that of EGFR (figure 3D, *right*; Pearson's correlation coeffcient=0.659, p<0.01), indicating that AMPK activation might be associated with EGFR expression in HNSCC.

### The effect of dasatinib on intracellular ATP, glucose, and PDK4 expression

**Figure 4:**
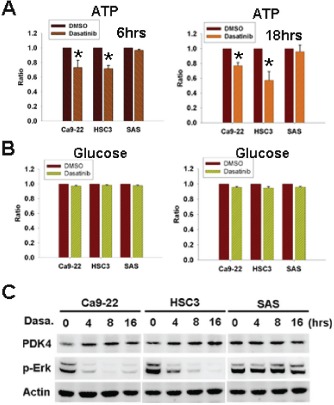
Dasatinib induced cellular ATP decrease and PDK4 up--regulation (A,B) The effect of 6-hr or 18-hr dasatinib (1uM) on cellular ATP (A) and glucose (B) levels. *, p<0.05. (C) The expression of PDK4 and p-Erk in HNSCC cells treated with dasatinib (1uM) for indicated intervals.

Glucose converted to pyruvate and undergone oxidative phosphorylation in the presence of oxygen to generate adenosine triphosphate (ATP) is the main source of cellular energy [23]. To investigate the mechanism of dasatinib-induced AMPK activation, intra-cellular ATP and glucose levels were examined. ATP was decreased by dasatinib in sensitive but not resistant cells (figure 4A). However, cellular glucose level was not affected (figure 4B). Pyruvate dehydrogenase (PDH) is a critical enzyme converting pyruvate to acetyl-CoA for ATP generation [24]. Its activity is inhibited by phosphorylation from pyruvate dehydrogenase kinase 4 (PDK4), leading to the decrease of pyruvate conversion to acetyl-CoA [25]. The transcription of PDK4 was reported to be repressed by Erk activation [25]. Our recent work showed the inhibition of Erk activation by dasatinib [20], so the expression of PDK4 was examined. PDK4 up-regulation associated with Erk inactivation in sensitive cells but not resistant cells was found (figure 4C), suggesting the involvement of PDK4 in ATP decrease and AMPK activation induced by dasatinib.

### Metformin potentiated anti-cancer effect of dasatinib

**Figure 5:**
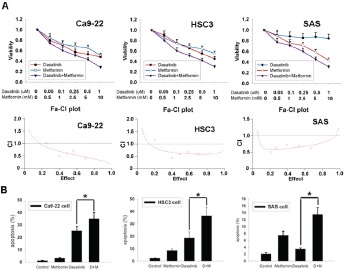
Metformin enhanced anti--cancer effect of dasatinib (A) The effect of dasatinib in combination with metformin for 48 hours at indicated doses on cellular growth inhibition. Cells were treated with dasatinib and metformin at a molar ratio of 1:10000. Growth inhibition was evaluated by MTT assay (*upper panel*). Dot, mean (n=3); bar, standard deviation. The combination index (CI) was calculated by median dose analysis (*lower panel*). CI smaller than one indicated synergism between dasatinib and metformin. (B) Metformin enhanced dasatinib-induced apoptosis. Cells were treated with dasatinib (1uM) in combination with metformin (10mM) for 48 hrs. Apoptosis was evaluated by subG1 analysis of fow cytometry. Column, mean (n=3); bar, standard deviation; *, p<0.05.

To evaluate the effect of AMPK activation on dasatinib-induced growth inhibition and apoptosis, an AMPK activator metformin was applied. Addition of metformin could increase dasatinib-induced growth inhibition in both sensitive and resistant cells (figure 5A, *upper panel*). The median effect analysis showed that the combination index (CI) was smaller than 1 (figure 5A, *lower panel*), indicating the synergism between dasatinib and metformin. Metformin also enhanced dasatinib-induced apoptosis in sensitive Ca9-22 and HSC3 cells and sensitized resistant SAS cells to dasatinib-induced apoptosis (figure 5B), suggesting that metformin could potentiate dasatinib-induced anti-cancer effect.

**Figure 6:**
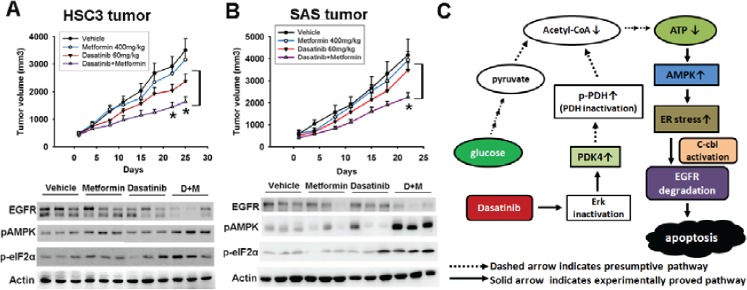
Figure 6: In vivo effect of metformin in combination with dasatinib in sensitive HSC3 tumor (A) and resistant SAS tumors (B) *Upper panel*, growth curve of xenograft tumors treated with indicated drugs. Dot, mean (n=8); bars, standard error; *, p < 0.05 by unpaired Student's t-test. *Lower panel*, the expression of EGFR, p-AMPK and p-eIF2α of HSC3 (A) or SAS (B) tumors. The representative three tumors from each treatment group at the end of experiments were analyzed by Western blotting. (C) Schematic illustration of dasatinib-induced AMPK activation, ER stress, and EGFR degradation.

To examine the in vivo effect of metformin in combination with dasatinib, sensitive HSC3 and resistant SAS cells were introduced into nude mice via subcutaneous administration. The mice with tumor xenografts reaching 100 mm^3^ were randomly divided in to 4 experimental groups, and treated with vehicle, metformin orally (400 mg/kg), dasatinib orally (60 mg/ kg/d) or in combination 5 days per week for 4 weeks. All mice tolerated this treatment well without significant toxicity and had stable body weights. Metformin enhanced dasatinib-induced anti-cancer effect of HSC3 (figure 6A, *upper panel*) or SAS tumors (figure 6B, *upper panel*). The tumor lysates were analyzed at the end of the experiment. Phosphorylation of AMPK and eIF2α and down-regulation of EGFR induced by dasatinib were enhanced by metformin in sensitive HSC3 tumors (figure 6A, *lower panel*). This event was also seen in resistant SAS tumors despite that dasatinib alone did not show any effect (figure 6B, *lower panel*). All the results indicated that activation of AMPK by metformin potentiated dasatinib-induced ER stress, EGFR degradation, and anti-tumor effect in vivo (figure 6C, schematic illustration).

## DISCUSSION

Few patients benefted from dasatinib in clinical trials despite consistent Src inhibition, implying that Src inhibition is not the determinant of dasatinib efficacy [19, 26]. Our recent work suggested that c-cbl-lysosome pathway was crucial for dasatinib-induced EGFR degradation and apoptosis in HNSCC [20]. We further reveal that ER stress could be a mechanism responsible for this effect, and activation of AMPK is shown to mediate dasatinib-induced ER stress and EGFR degradation. Addition of metformin could enhance the anti-cancer effect of dasatinib in both sensitive and resistant cells in vitro and in vivo. Our study suggests that AMPK-dependent ER stress plays a critical role in dasatinib-induced EGFR degradation and apoptosis. The anti-cancer effect of dasatinib can be improved by disclosing this new mechanism.

Metformin is the most widely used drug for the treatment of type 2 diabetes. It can inhibit respiratory chain of mitochondria leading to energy restriction and AMPK activation [27-28]. AMPK activation inactivates mammalian target of rapamycin (mTOR) pathway to inhibit cell growth and proliferation [29]. AMPK activator resveratrol has been reported to inhibit growth of imitinib-resistant chronic myeloid leukemia (CML) cells by mTOR inhibition [30]. Therefore, metformin is anticipated to have anti-cancer effect. Epidemiological studies suggest that metformin lowers the risk of cancer and improve cancer prognosis [8, 31-32]. Its prevention on HNSCC tumor development has been reported to be through mTOR inhibition by causing cell cycle arrest and inhibiting protein translation [33-34]. Our data also showed that metformin induced growth inhibition and apoptosis. In vivo study revealed that metformin induced ER stress and inhibited tumor growth. Therefore, inhibition of protein translation, a common consequence of both ER stress and AMPK-mTOR inhibition, might be an important anti-tumor mechanism of metformin. Stemness of cancer cells is a mechanism of resistance to anti-cancer treatment [35]. Metformin has been reported to enhance trastuzumab efficacy in animal model by killing CD44^+^/CD24^−^ breast cancer stem cells [35-36]. It also inhibits PIK3CA mutation-driven breast cancer cells in vivo [37]. Given that metformin is a FDA approved drug, its mechanism of activity deserves further investigation.

Recently, dasatinib has been reported to inhibit cellular ATP in sensitive but not resistant chronic lymphocytic leukemia (CLL) cells [38]. This differential effect is interpreted by AMPK-mediated metabolic programming between oxidative phosphorylation and aerobic glycolysis [38]. Our data also showed that dasatinib induced AMPK activation and ATP decrease without alteration of glucose. Up-regulation of PDK4 could interpret this effect. The link of Erk/PDK4/PDH/ ATP suggests the association between growth factor and metabolic pathway. Activation of growth factor receptor pathway might facilitate nutrient uptake for cell growth and proliferation. For example, EGFR has been reported to involve in glucose transport by stabilizing the active glucose transporter, sodium-glucose linked transporter 1 (SGLT1) [39]. Activation of glycogen synthase kinase 3(GSK-3)-mTOR pathway increases the expression of glucose transporter to facilitate glucose uptake [40]. Furthermore, our results suggested that EGFR degradation was attributed to Erk inactivation-induced PDK4 up-regulation to decrease ATP and activate AMPK, providing the clue that AMPK activation might enhance anti-cancer of dasatinib through targeting EGFR. Metformin in combination with dasatinib further activated AMPK and induced EGFR degradation as well as apoptosis, implicating that induction of metabolic stress could mediate anti-tumor effect and serve as an anti-cancer strategy. Targeting cellular metabolism by metformin in combination with everolimus, a mTOR inhibitor, has been reported to sensitize chemotherapy against breast cancer cells [41]. mTOR inhibitor rapamycin in combination with metformin could also induce G1 and G2 arrest to protect normal fibroblast from the toxicity of mitotic inhibitor palcitaxel [42]. Therefore, metformin plus mTOR inhibitor might be a rational strategy to treat cancer. Clinical trials of metformin in combination with everolimus or temsirolimus are ongoing (NCT01529593 and NCT01627067, http://clinicaltrials.gov).

In addition to translational inhibition, protein degradation is a compensatory response to ER stress [2]. Proteins designated for degradation by classical ER-associated protein degradation (ERAD) pathway are ubiquitinated and subsequently degraded by proteasome [43]. However, c-cbl-mediated ubiquitination, endocytosis, and lysosome degradation is the most elucidated pathway for EGFR degradation [44]. Our study showed that ER stress induced EGFR down-regulation which was reversed by c-cbl knockdown. C-cbl activity and the association of c-cbl-EGFR were increased by ER stress. Furthermore, lysosome inhibitor NH_4_Cl attenuated ER stress-induced EGFR degradation. These results suggested that ER stress-induced EGFR degradation is c-cbl-dependent and might be through lysosome. The extent of protein ubiquitination might affect the route of degradation that monoubiquitination mediates receptor endocytosis and lysosome degradation while polyubiquitination targets proteins for proteasomal degradation [45-46]. Proteasome has also been shown to degrade EGFR [47-48]. It is possible that the extent of EGFR ubiqitination determines the its subsequent lysosome or proteasome degradation during ER stress.

The effect of AMPK activation on ER stress induction is controversial. Activation of AMPK leads to ER stress and exerts anti-cancer effect in leukemia cells [10], and phenformin induces ER stress through AMPK activation in HCC cells [11]. Glycolysis inhibition by 2DG activates AMPK and ER stress in pancreatic cancer cells [49]. Our previous work also shows that statin induces ER stress through AMPK activation [22]. In contrast, activation of AMPK inhibits ER stress to protect cardiomyocyte against hypoxic injury and attenuate atherosclerosis in vascular endothelial cells [50-51]. We found that knockdown AMPK attenuated dasatinib-induced ER stress. Activation of AMPK by 2DG or AICAR also induced ER stress, suggesting that activation of AMPK might induce ER stress per *se* and enhance dasatinib-induced ER stress. The association of AMPK activation and ER stress might be dependent on cellular context (cancer or differentiated cells).

We found that AMPK knockdown up-regulated EGFR expression, and that AMPK activation by 2DG or AICAR decreased EGFR expression. Activation of AMPK by quercetin, an anti-oxidant flavonoid, has also been reported to induce EGFR down-regulation [52]. These results imply that AMPK activation might decrease EGFR expression. However, the expression of phosphor-AMPK (p-AMPK) but not AMPK was positively correlated with EGFR in HNSCC cells and human specimens, suggesting that baseline AMPK activation was in concordance with EGFR expression. The effect of baseline and pharmacologic activation of AMPK on EGFR expression seems contradictory and deserved further studies. Given that EGFR is an oncoprotein and correlates with poor outcome [14], the clinical relevance of AMPK activation needs to be further clarifed.

In conclusion, our study revealed that AMPK-dependent ER stress is the determinant of dasatinib-induced anti-cancer effect. Further activation of AMPK by metformin might enhance dasatinib anti-cancer effect in HNSCC.

## MATERIAL AND METHOD

### Ethical statement

Animal study was approved by National Taiwan University College of Medicine and College of Public Health Institutional Animal Care and Use Committee (project number: 20110395).

Human study was approved by the institutional review board of Far-Eastern Memorial Hospital (FEMH-IRB-099083-E).

### Cell culture

Ca9-22 was provided by Dr. Hsin-Ming Chen (Graduate Institute of Oral biology, College of Medicine, National Taiwan University) in 2010. SAS was provided by Dr. Han-Chung Wu (Institute of Cellular and Organismic Biology, Academia Sinica) in 2010. HSC3 was provided by Dr. Kwang-Yu Chang (National Health Research Institutes) in 2010. Ca9-22 and SAS cells are cultured in Dulbecco's modified Eagle's medium. HSC3 cells are cultured in minimal essential medium. Culture medium is added with 0.5 μg/ml hydrocortisone, and 10% fetal bovine serum. Cells were incubated in a 37°C humidified incubator under an atmosphere of 5% CO2 in air.

### Materials

Dasatinib (Sprycel^®^) was kindly provided by Bristol-Myers Squibb pharmaceuticals. 4-phenyl butyric acid (PBA), brefeldin-A, tunicamycin, NH_4_Cl, compound C, 2-DG, AICAR, and metfomin were purchased from Sigma-Aldrich. All experimental drugs were dissolved in DMSO (Sigma Chemical Co). Anti-EGFR, AMPK, eIF2μ, CHOP, and actin are purchased from Santa Cruz Biotechnology. Anti-phospho-eIF2μ and phosphor-AMPK are purchased from Cell Signaling.

### Cell Viability Assay

Cell viability is determined using the MTT assay. Cells were plated in triplicate in 96-well plates and treated with increasing concentrations of dasatinib. After 48 hours of incubation, cell growth was measured using 0.5mg/ ml 3-(4,5-dimethylthiazol-2-yl)-2,5-diphenyltetrazolium bromide (Sigma, St. Louis, MO) colorimetric method. The blue MTT formazan precipitate was then dissolved in 100 μL of DMSO. The absorbance at 550 nm was measured on a multi-well plate reader. Cell viability was expressed as a percentage of control. Data are shown as the mean value ± standard error of the mean of three independent experiments.

### Calculation of synergism

The medium-effect method was used to analyze dose-response data for single drug or multiple drugs. The synergistic effect of multiple drugs was determined by the defnition of Chou and Talalay [53]. The Chou and Talalay combination index (CI), a well-established index refecting the interaction of two drugs, was calculated at different levels of growth inhibition with the use of software package Calcusyn (Biosoft, Cambridge, UK). The CI for 50% growth inhibition (IC_50_) was calculated as follows:

CI values of <1, 1, and >1 indicate synergistic, additive, and antagonistic effects, respectively.

### Western immunoblotting

Following treatment with specific drugs, total cell lysates are prepared and subjected to SDS-PAGE using 7.5% or 10% running gels. Western blotting was done as previously described [20].

### Co-immunoprecipitation assay

Cells were harvested and lysed on ice for 30 min in lysis buffer (50 mM Tris-HCl, pH 7.4, 100 mM NaCl, 0.5% Nonidet P-40, 50 mM NaF, 1 mM Na3VO4, 5 mM sodium pyrophosphate, and a protease inhibitor tablet). The cell lysates were centrifuged at 14,000*g*for 15 min, and the supernatants were recovered. Supernatants containing equal amounts of proteins were incubated with 2.5 mg of primary antibodies overnight at 4 °C. The immunoprecipitates were harvested using protein G PLUS-agarose beads (Santa Cruz Biotechnology) that were washed once with regular washing buffer (50 mM Tris-HCl, 100 mM NaCl, 1 mM EDTA, and 0.5% Nonidet P-40), twice with high salt washing buffer (50 mM Tris-HCl, 500 mM NaCl, 1 mM EDTA, and 0.5% Nonidet P-40), and another time with regular washing buffer. Immunoprecipitates were then eluted by boiling the beads for 5 min in SDS/PAGE sample buffer and characterized by Western blotting.

### Flow cytometry

Cell cycle and apoptosis were evaluated by flow cytometry. After treated with specific agents, HNSCC cells are trypsinized, washed in PBS and centrifuged at 1000 rpm for 5 mins. Then the cells are fixed with 75% ethanol and freezed in -20⁪. After washing with PBS twice, the cells are suspended in 500 μl solution containing propidium iodide and RNAase. Flow cytometry was performed using BD FACS Calibur cytometer with Cellquest software. Apoptosis is determined by subG1 ratio of cell cycle analysis.

### Glucose measurement assay

Intracellular glucose was measured by flow cytometry with 2-[N-(7-Nitrobenz-2-Oxa- 1,3-Diazol-4-yl)Amino]- 2-Deoxy-D-Glucose (2-NBDG). Cells were treated with specific drugs for indicated time, and 100uM 2-NBDG was added to cell medium 15 minutes prior to flow cytometry analysis.

### ATP measurement assay

Intracelluar ATP level is measured according to manufacturer's instruction (Perkin-Elmer). Briefly, cells are grown in 96-well plate and treated with dasatinib for indicated time. At the end of treatment, lysis buffer and substrate solution are added to cell medium. Cells are placed at orbital shaker at 700rpm for 5 minutes. Luminescence of cells is measured by luminometer, and ATP concentration is determined by comparison with standard curve. ATP level is further standardized to protein concentration.

### Live cell imaging

Cells were co-transfected with EGFR-RFP and c-cbl-GFP for 48 hours. ASTEC cultured cell monitoring system and Leica DM IRE2 inverted research microscope were used for live cell imaging according to manufacturer's instruction. Live cells treated with drugs were recorded at indicated time. The co-localization of EGFR and c-cbl was further calculated by Zen software (Carl Zeiss).

### siRNA knockdown analysis

AMPK, PERK, and c-cbl SmartPool and control siRNA (Dharmacon) are transfected with Lipifectamine 2000 according to the manufacturer's instructions. Briefly, 50% confluent cells in 6-well plate are transfected with 100 pmol siRNA in 1 mL of serum-free medium for 6 h at 37°C. Then, 1 mL of medium containing 20% FBS is added to the transfection mixture. After 24 h, cells are treated with dasatinib for 24h. The cells were lysed and the protein expression was analyzed by western blot.

### Immunohistochemical staining and the scoring of p-AMPK and EGFR from HNSCC specimens

Surgical resected HNSCC tumor specimens were collected after the informed consents obtained from patients. The study was approved by the institutional review board of Far-Eastern Memorial Hospital (FEMH-IRB-099083-E). Written informed consent was obtained from all patients. Formalin-fxed colonic tissues from mice were embedded in paraffn by routine procedures. H&E-stained, 4-μm sections of major organs were evaluated microscopically by a pathologist. The expression of relevant biomarkers in representative sections of tumor tissues was detected by immunohistochemical staining using specific primary antibodies. The IHC score of EGFR was calculated with the formula: 1 × (percentage of cells staining weakly [1 +]) + 2 × (percentage of cells staining moderately [2 +]) + 3 × (percentage of cells staining strongly [3 +]) [54]. The IHC score of p-AMPK was applied from that proposed by Nardo et al [55]. The proportion of expression was given scores 1 to 6 (0%– 4%=1, 5%–20%=2, 21%–40%=3, 41%–60%=4, 61%– 80%=5, and 81%–100%=6). The intensity of p-AMPK was scored as negative, 1+, 2+, and 3+. The IHC score of p-AMPK was calculated by multiply the intensity and the proportion of cells staining. Each pathological specimen was reviewed for ten high-power filed by experienced pathologists.

### Subcutaneous ectopic xenograft tumor model

Sixty-four female NCr athymic nude mice (4 weeks of age) were obtained from the National Laboratory Animal Center (Taipei, Taiwan). At the age of 6 weeks old, HSC3 cells (n=32, 4×10^6^/mice) and SAS cells (n=32, 2×10^6^/mice) were inoculated subcutaneously in the rear left flank. Cells were suspended in 0.1 ml PBS and mixed with 0.1 ml matrigel. Dasatinib and Metformin were dissolved in citrate buffer (pH=3.1) and PBS, respectively. When tumors reached 100 mm^3^, HSC3-bearing or SAS-bearing mice were randomized to take vehicle, metformin (400mg/kg/d), dasatinib (60mg/kg/d), or in combination 5 days per week for 4 weeks. Tumor volume is calculated using the formula V (mm^3^) = [ab^2^]/2, where a is the length and b is the width of the tumor.

### Statistical analysis

Quantitative data are presented as means ± standard deviation (SD) from 3 independent experiments. In animal study, tumor growth data are reported as mean tumor volume ± SE. The significance of differences was evaluated with 2-tailed Student's t-test. P > 0.05 was considered statistically significant. The SPSS software (Windows version 18) was used for statistical analysis.

## Supplementary Figures and Tables






