# Correction: Metformin sensitizes anticancer effect of dasatinib in head and neck squamous cell carcinoma cells through AMPK-dependent ER stress

**DOI:** 10.18632/oncotarget.26997

**Published:** 2019-05-28

**Authors:** Yu-Chin Lin, Meng-Hsuan Wu, Tzu-Tang Wei, Yun-Chieh Lin, Wen-Chih Huang, Liang-Yu Huang, Yi-Ting Lin, Ching-Chow Chen

**Affiliations:** ^1^ Graduate Institute of Pharmacology, National Taiwan University College of Medicine;; ^2^ Department of Oncology, National Taiwan University Hospital;; ^3^ Department of Internal Medicine and; ^4^ Department of Pathology, Far-Eastern Memorial Hospital;; ^*^ Equal contribution to work

**This article has been corrected:** The correct Figure 3 is given below.

About your inquiry on July 12, 2018, related to the paper “ Metformin sensitizes anticancer effect of dasatinib in head and neck squamous cell carcinoma cells through AMPK-dependent ER stress” published in Oncotarget 2014, Volume 5, Number 1, National Taiwan University (NTU) have finished the investigation which is described below:

1. The Procedure of Investigation:

We formed an Investigation Committee right after we received your inquiry. Both the first (Yu-Chin Lin) and the corresponding (Ching-Chow Chen) authors provided their original gel images and lab notebooks in written and attended our committee meeting on October 26, 2018. The committee evaluated the provided information, judged on their personal statement/reply, discussed this case thoroughly, and then made the final conclusion.

2. Investigation Conclusion:

NTU concluded that the revised New Figure 3 submitted on January 4, 2017 indeed contained uncorrected panels which were existed in the original published version. While the first author (Yu-Chin Lin) corrected the repeatedly used in the left part of the panel 3B (which is identical to the actin image in the original panel 3D) on January 4, 2017, he did not realize that the right part of the panel 3B was used in the left part actin image of panel 3C, which was existed in the original published version and the revised version as well.

**Figure 3:**
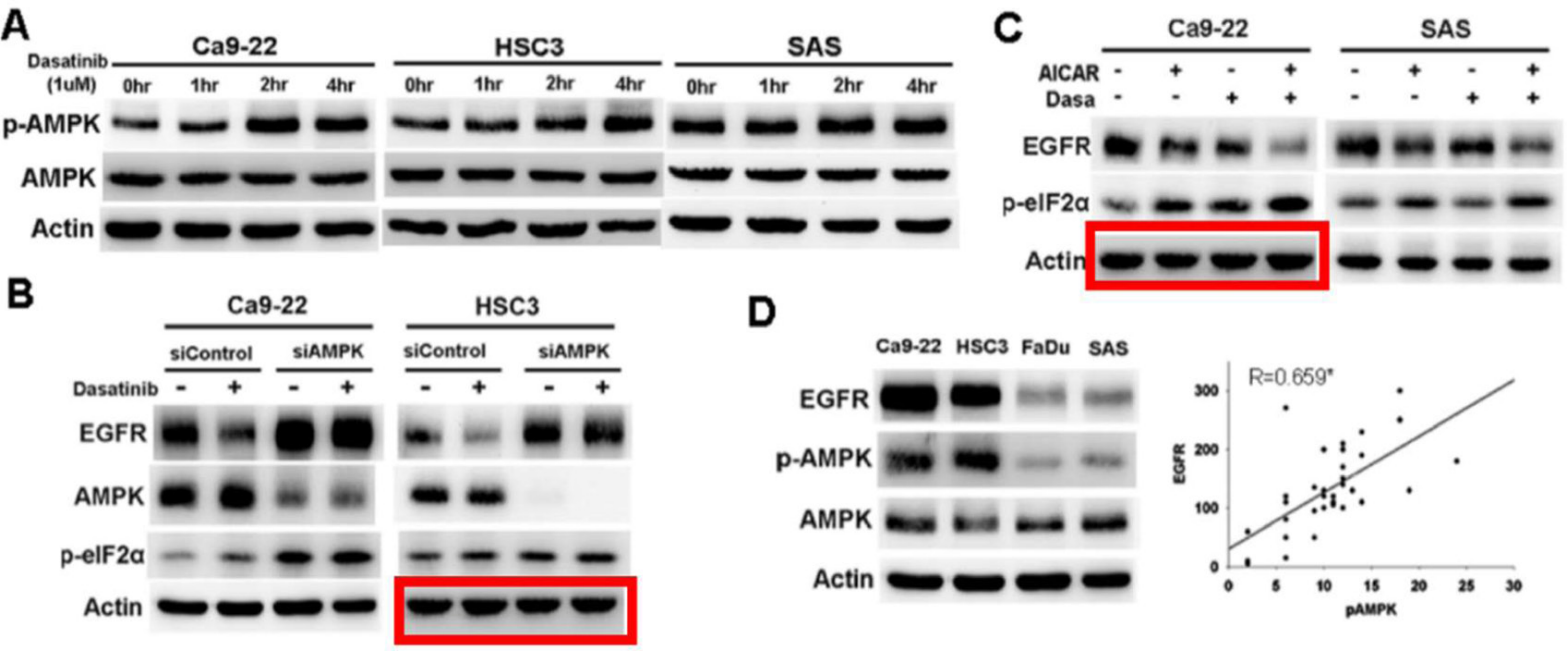
AMPK activation mediated dasatinib-induced ER stress and EGFR degradation.

**Figure 3:**
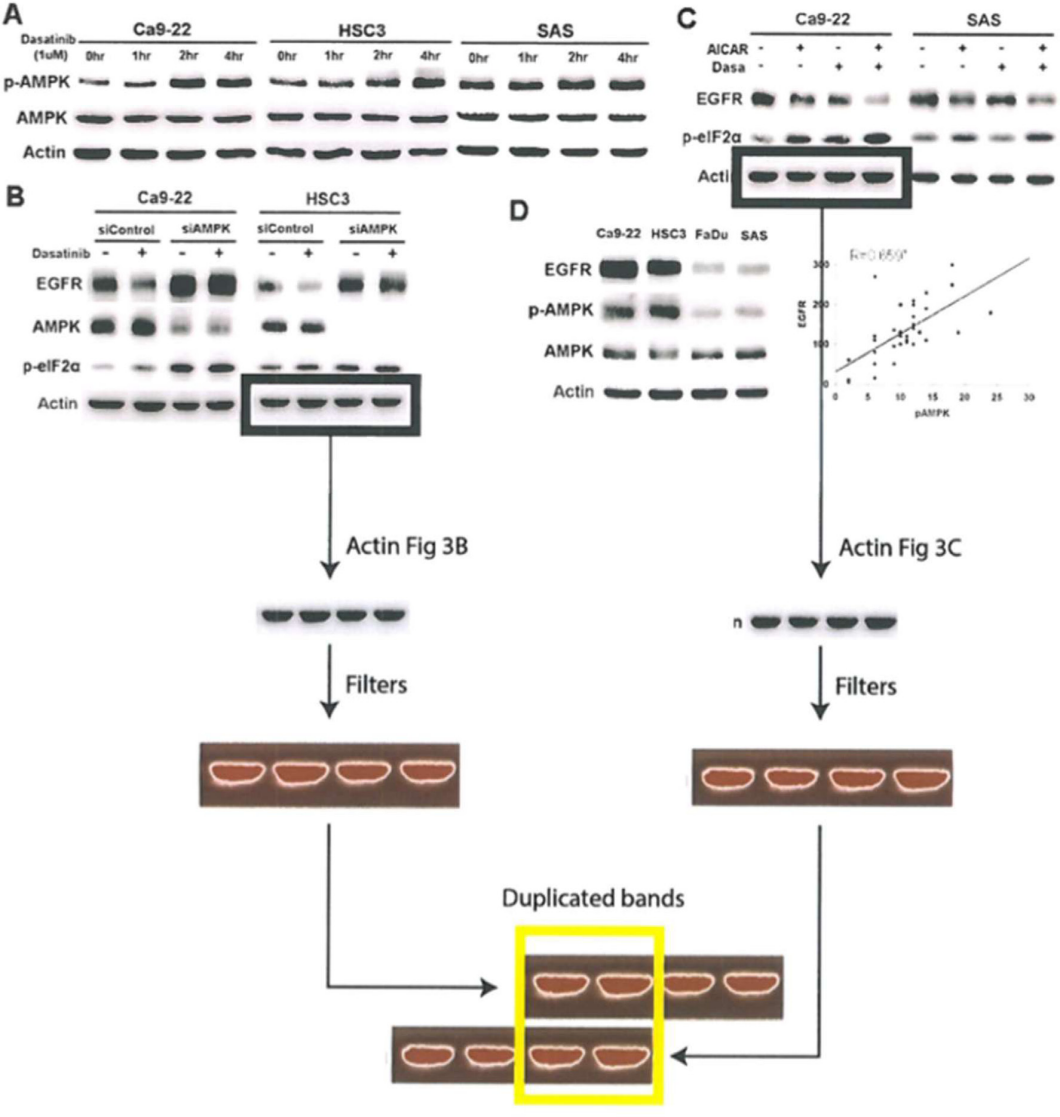
AMPK activation mediated dasatinib-induced ER stress and EGFR.

On January 10, 2017 they did try to revise again with the correct original data of panel 3B and 3C that the Investigation Committee confirmed during the investigation. But, due to the mistakes that they made in the revision on January 4, 2017, the Investigation Committee felt that the first author played the major role for the sloppiness, and the corresponding author did not fulfill her role of supervision.

Therefore, NTU issued the corresponding author, Ching-Chow Chen, a warning in written because of this mistake, and told her that, as a corresponding author, she should be more careful to avoid similar issues occurring again in the future. As to the first author, Yu-Chin Lin, NTU will inform the conclusion of the investigation to his current institute (Mennonite Christian Hospital) because he does no longer work at NTU.

The authors declare that these corrections do not change the results or conclusions of this paper.

Original article: Oncotarget. 2014; 5:298–308. 298-308 . https://doi.org/10.18632/oncotarget.1628

