# Correction: Metformin sensitizes anticancer effect of dasatinib in head and neck squamous cell carcinoma cells through AMPK-dependent ER stress

**DOI:** 10.18632/oncotarget.27228

**Published:** 2019-10-01

**Authors:** Yu-Chin Lin, Meng-Hsuan Wu, Tzu-Tang Wei, Yun-Chieh Lin, Wen-Chih Huang, Liang-Yu Huang, Yi-Ting Lin, Ching-Chow Chen

**Affiliations:** ^1^ Graduate Institute of Pharmacology, National Taiwan University College of Medicine, Taipei, Taiwan, Republic of China; ^2^ Department of Oncology, National Taiwan University Hospital, Taipei, Taiwan, Republic of China; ^3^ Department of Internal Medicine and; ^4^ Department of Pathology, Far-Eastern Memorial Hospital,Taipei, Taiwan, Republic of China

**These articles have been corrected:** The proper images for Figure 3 and Figure 4 are shown below. The authors declare that these corrections do not change the results or conclusions of this paper.

Original article: Oncotarget. 2014; 5:298–308. 298-308. https://doi.org/10.18632/oncotarget.1628

**Figure 3:**
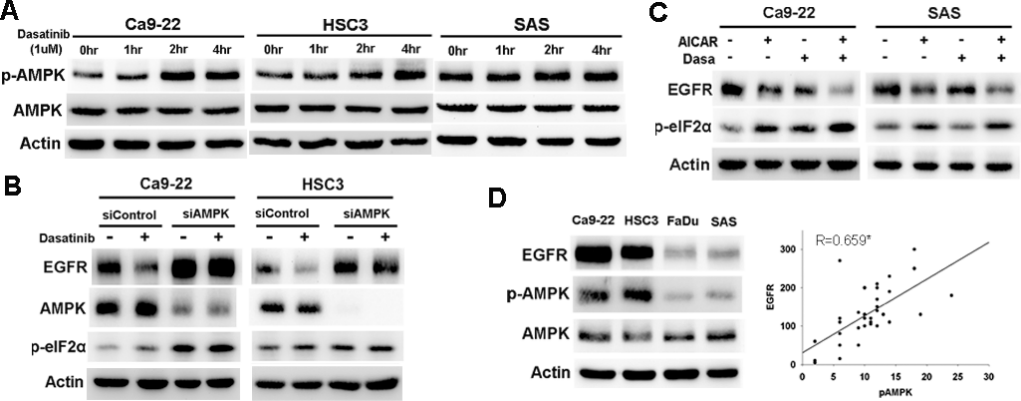
AMPK activation mediated dasatinib-induced ER stress and EGFR degradation. (A) The effect of dasatinib on AMPK activation. Cells were treated with dasatinib (1uM) for indicated intervals. The expression of p-AMPK and AMPK was evaluated. (B) The effect of AMPK knockdown on dasatinib-induced EGFR degradation and ER stress. Cells were treated with control or AMPK siRNA and then with dasatinib for 24 hours. (C) The effect of AMPK activation on dasatinib-induced EGFR degradation. Cells were treated with dasatinib with or without AICAR (10uM) for 24 hours. The expression of EGFR p-eIF2α, and AMPK was evaluated. (D) The correlation between p-AMPK and EGFR expression. *Left*, the expression of EGFR, p-AMPK, and AMPK in HNSCC cells. *Right*, the correlation of p-AMPK and EGFR expression in resected human specimens. Pearson’s correlation coefficient=0.659; *, p<0.01.

**Figure 4:**
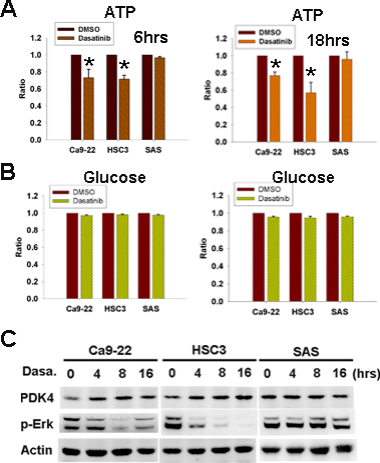
Dasatinib induced cellular ATP decrease and PDK4 up-regulation. (A,B) The effect of 6-hr or 18-hr dasatinib (1uM) on cellular ATP (A) and glucose (B) levels. *, p<0.05. (C) The expression of PDK4 and p-Erk in HNSCC cells treated with dasatinib (1uM) for indicated intervals.

