# Supplementary figures and images for: Metformin sensitizes anticancer effect of dasatinib in head and neck squamous cell carcinoma cells through AMPK-dependent ER stress
Source: Oncotarget. 2014 Jan 7;5(1):298–308. doi: 10.18632/oncotarget.1628 (PMC3960210; doi:10.18632/oncotarget.1628)

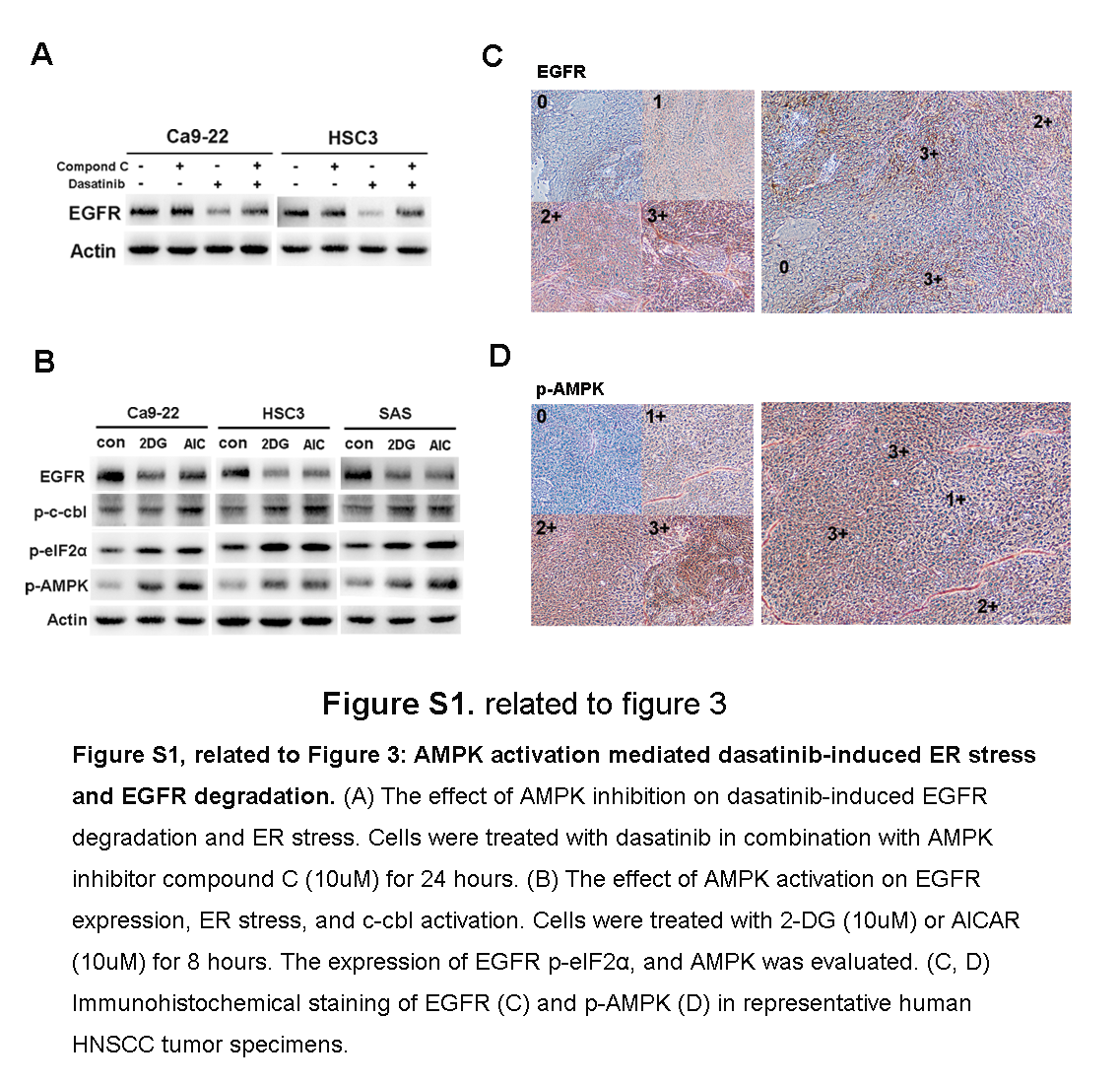

Supplement: Supplementary file 1 [file oncotarget-05-0298-s001.tif]
